# A Matrix-Based Proactive Data Relay Algorithm for Large Distributed Sensor Networks

**DOI:** 10.3390/s16081300

**Published:** 2016-08-16

**Authors:** Yang Xu, Xuemei Hu, Haixiao Hu, Ming Liu

**Affiliations:** School of Computer Science and Engineering, University of Electronic Science and Technology of China, Chengdu 611731, China; huxuemei1990@126.com (X.H.); 201511060118@std.uestc.edu.cn (H.H.); mingliu.uestc@gmail.com (M.L.)

**Keywords:** proactive data relay, information fusion, large distributed sensor networks, matrix-based computing

## Abstract

In large-scale distributed sensor networks, sensed data is required to be relayed around the network so that one or few sensors can gather adequate relative data to produce high quality information for decision-making. In regards to very high energy-constraint sensor nodes, data transmission should be extremely economical. However, traditional data delivery protocols are potentially inefficient relaying unpredictable sensor readings for data fusion in large distributed networks for either overwhelming query transmissions or unnecessary data coverage. By building sensors’ local model from their previously transmitted data in three matrixes, we have developed a novel energy-saving data relay algorithm, which allows sensors to proactively make broadcast decisions by using a neat matrix computation to provide balance between transmission and energy-saving. In addition, we designed a heuristic maintenance algorithm to efficiently update these three matrices. This can easily be deployed to large-scale mobile networks in which decisions of sensors are based on their local matrix models no matter how large the network is, and the local models of these sensors are updated constantly. Compared with some traditional approaches based on our simulations, the efficiency of this approach is manifested in uncertain environment. The results show that our approach is scalable and can effectively balance aggregating data with minimizing energy consumption.

## 1. Introduction

Large distributed sensor networks have been widely used in both military and civilian applications [1], such as target tracking [2], disaster response [3] and field surveillance [4]. In these applications, although each sensor is able to get some crude information, the data is usually imprecise or noisy with very low fidelity. As a consequence, the data in this form cannot be directly used for automatic planning or supporting human decisions, and has to be fused with other relevant data [5].

To fuse distributed data in a network, the key is to relay multiple-source data and aggregate enough amount of data to a single node in order to achieve high confidence information fusion [6]. However, with the progress of the development of state-of-the-art mobile sensor applications, networks emerge with new characteristics that pose challenges to existing information fusion approaches. The astronomical growth of networks involving thousands of sensors is a typical challenge in this respect. In these large networks, no single sensor can respond as the center and gain complete statistic of states of the entire network. In addition, because these networks are dynamically changing due to the mobility of sensors, or movements as a result of the surrounding air or ocean currents [7], node failures are not rare. Additionally, most sensors in these large networks have lightweight processing units, which make high constraint of energy consumption ideal for their operations.

Following the importance of information fusion, many data relay protocols have been developed to diffuse data in sensor networks. Flooding [8] adopts a straightforward protocol for sensors to rebroadcast any new data received, but suffers from the intrinsic excessive energy consumption and network congestion due to the large number of duplicated messages. Traditional hierarchical [8] or centralized routing [9,10,11,12] approaches, which are designed for fixed structure or where only sink nodes respond to information fusion are not also ideal for data fusion in mobile sensor networks. The predefined backbone nodes in these approaches will exhaust their power very quickly because of the heavy transmission burden and enormous cost on routing maintenance. To avoid these problems, many other distributed approaches have been developed for cases whereby all nodes can be treated as sink nodes. Those approaches include Information via Negotiation(SPIN) [13], Scalable Broadcast Algorithm (SBA) [14], The Lightweight and Efficient Network Wide Broadcast (LENWB) [15], Dominant Pruning [16] and Dynamic Probabilistic Flooding Algorithm [17]. Even though these approaches can guarantee a high quality information fusion, not all sensors would need all the data because any piece of information can only be fused by the sensor that aggregates enough relative data first. Therefore, the unnecessary data coverage may end with huge amount of energy consumption and redundant communication cost. SPIN can address this limitation to some extent by negotiating before broadcast, and allows sensors to transmit only data that others would need. Unfortunately, it is a reactive protocol, and a large number of negotiated messages makes it time-consuming and cost-ineffective for distributed data fusion. Some algorithms [18] can proactively relay data by forecasting the needs before a given set of data is broadcast. For example, a sensor would rather like to rebroadcast the data that is relative to the data of its neighbors only. However, when sensors cannot gain a complete view over an entire network, an intelligent proactive algorithm could be very difficult to implement [19].

In this paper, we present a novel matrix-based energy-saving and proactive algorithm to relay data in large distributed sensor networks. To deliver the core competence of this approach, we employ no querying process, and each node proactively forwards data to its neighbors by estimating the needs of neighbors based on its local knowledge about itself and the neighbors. From this viewpoint, we encode local knowledge of sensors into three neat matrices: local connection matrix (C); local data distribution matrix (D); and utility matrix (U) to encode utility of adding data into a data set. With these matrices, a compact and light-weight algorithm is proposed, where each node makes a series of simple all-in-one matrix computation to evaluate the benefits and cost of actions. Comparing the benefits against cost allows serious decisions to be made whether a piece of sensor data should be broadcast to make a good tradeoff between data relay to get high quality information and prolonging the lifetime of the network. In addition, energy consumption in data relay is taken into consideration towards cautious decision-making processes. As sensors do not have global knowledge of the network, a heuristic algorithm is proposed to maintain the size and value of these matrices from incoming messages to adapt to sensor mobility. By introducing matrix computations, and as our matrices only store local knowledge and updated in time, our approach is lightweight and can easily be deployed in large mobile sensor networks. Further, to manifest feasibility of this approach, we discuss the simulations carried out, and the results show that our approach can perform well in dynamic environments to effectively balance aggregating data with minimizing energy consumption.

## 2. Related Work

Many data relay protocols have been designed for distribute data fusion in wireless sensor network [20,21,22]. They can be categorized as two groups according to where the fusion occurs.

One strand of protocols are based on fix or predefined sink nodes. They are designed for multiple sensors to forward data to specific sink nodes for data fusion, such as Directed Diffusion [9], in which a sink floods its interests to build reverse paths from all potential sources to the sink. Rumor Routing [10], Constrained anisotropic diffusion routing (CADR) [11] and GRAdient Broadcast [12] are some variations of Directed Diffusion. In Rumor routing, sink floods queries while sensors flood events which make Rumor routing performs better than Directed Diffusion when number of events is small. CADR introduces an information utility measure to select which sensors query and dynamically guide data routing. GRAB builds and maintains a cost field to provide sensors the direction to forward sensing data. It is a robust data delivery algorithm addressed nodes failures and link failures. However, since these protocols are based on single-gateway architecture that makes them not fit for large scale mobile sensor network [21]. First of all, sensors near sink nodes have heavy burden to relay data and their energy will be exhausted in short time. Second, sensors are typically not capable of long-haul communication and the latency in communication can not be ignored. Third, energy on finding ways to sink nodes is huge which makes them not suitable for mobile networks where network topology dynamically changes.

To allow the system to be able to cover a large area of interest without degrading the service, networking clustering has been pursued in some routing approaches. LEACH [21] and LEACH series [23] are hierarchical routing algorithms for sensor networks. Cluster heads change randomly over time in order to balance the energy dissipation of nodes. Each node transmits directly to the cluster-head. They are completely distributed and requires no global knowledge of network. However, they use single-hop routing where each node transmits directly to the cluster-head and the sink. Therefore, it is not applicable to networks deployed in large regions . Further more, the idea of dynamic clustering brings extra overhead, e.g. head changes, advertisements etc., which may diminish the gain in energy consumption.

For the second strand of protocols, there is no predefined sink nodes, sensors are teated equally as probable sink nodes, and data fusion can be done by any sensor unless it aggregates enough relative data. The most straight forward protocol is flooding [8], in which sensors rebroadcast any new data it receives. Apparently, FLOODING consumes too much energy on the transmission of redundant data. To reduce the redundant data of flooding, some reactive and proactive protocols are proposed. Sensor Protocols for Information via Negotiation(SPIN) [13] is a reactive protocol which avoids redundant data transmission by meta-data negotiation with neighbors. sensors only forward data to the neighbors that need it. However, it is infeasible for distributed fusion where the size of meta-data is close to useful data because the frequent query data transmission is not cost-effective and introduces non-negligible time delay.

Proactive protocols do not need queries to avoid redundant data transmission but proactively decide if rebroadcasting data received based on the local topology and static information of redundant data, such as Scalable Broadcast Algorithm (SBA) [14], The Lightweight and Efficient Network Wide Broadcast (LENWB) [15], Dominant Pruning [16] and Dynamic Probabilistic Flooding Algorithm [17]. SBA and Dominant Pruning maintain the local network topology by hello messages. In SBA, sensors only rebroadcast the data that at least one of its neighbors do not know. In Dominant Pruning, rebroadcasting nodes proactively choose one or more of its neighbors as rebroadcasting nodes by Greedy Set Cover algorithm. In Dynamic Probabilistic Flooding Algorithm, sensors obtain neighbor information by basic flooding and divide neighbors into three types: parent (upper level), sibling (same level), and child (lower level) nodes. A node with the more children nodes and less siblings needs the lower retransmission probability. These three protocols try to cover all the nodes with all the data to guarantee at least one node aggregates enough relative data to fuse into information. However, in large sensor network, too much energy is consumed on unnecessary coverage of such a huge number of sensor nodes, for a piece of information is only needed to be fused by one sensor. In addition, in mobile sensor network, too much energy is consumed on the transmission of hello message to maintain local topology.

## 3. Problem Description

**Figure 1:**
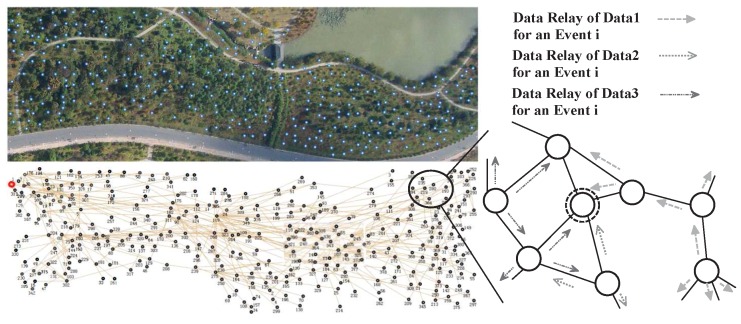
An overlook on the large-scale sensor network deployment and the real topology.

A typical scenario of a large scale mobile sensor domain is illustrated in Figure 1, where distributed sensors are randomly deployed for remote operations in a large unstructured geographical area to detect events. For their limited communication ranges and wide distribution, each of them can only relay data to a few of the others directly. But for the low quality of those sensor readings, data has to be transmitted until a single node get enough relevant data to produce high confident information, which denoted as a double circled sensor in the figure.

Let G(V,E) be the topology graph of this network. *V*=$\left\{v_{1},v_{2},...,v_{i},...\right\}$ denotes the set of mobile sensor nodes such as sonics, microwave, infrared and x-ray sensors. $E=\{e_{ij}|\forall v_{i},v_{j}\in V,P\left(v_{i},v_{j}\right)>0\}$ consists of edges between any two sensors, where $P(v_{i},v_{j})$ is the connection probability between two sensors. Supposed that each sensor has an identical communication range *r*, $P(v_{i},v_{j})$ can be calculated according to the channel propagation model [24,25]: $P\left(v_{i},v_{j}\right)=\left(1-f\left(d_{ij},1/r\right)\right)$ where $d_{ij}$ is the distance between $v_{i}$ and $v_{j}$. Based on *E*, the neighbor set $N(v_{i})$ of each sensor $v_{i}\in V$ is $\left\{v_{j}|\forall v_{j}\in V,e_{ij}\in E\right\}$.

Let us consider a case of sensors deployed to track multiple stationary or moving targets $Target=\{T_{1},T_{2},...,T_{m}\}$. It is possible that a given target $T_{k}$ can be detected by multiple sensors at the same time. For example, when a hostile vehicle is moving through a given intersection, a shock sensor detects the vibration when it passes and an infrared sensor nearby receives the infrared signal from the vehicle. After detecting a target, sensor $v_{i}$ will analyze the raw data(vibrates, infrared signal and so on), and generates a data $d_{j}$ about this target. Data $d_{j}$ can be denoted as a tuple <sourceID,identity,location,timestamp,path>:

- sourceID is the ID of the sensor that senses this data.
- identity is the confidence hypotheses about the target identities $T_{k}$, which can be expressed as $cd_{j}=\left\{c_{d_{j}}\left(T_{1}\right),c_{d_{j}}\left(T_{2}\right),...,c_{d_{j}}\left(CLUTTER\right),\right\}.$ An example is shown in Table 1.
- location is the geographical location of the target when detected.
- timestamp is the system time when the target is detected.
- path records sensors that pass this data.

The location and timestamp of a target are used to determine if two pieces of data are referring to the same target. If that is the case, they are called relevant. Since the data from a sensor is always noisy, uncertain and cannot be used directly for automatic planning or supporting human decisions, data should be relayed around the network to meet more relevant data to produce a higher quality information [26].

The basic distributed data relay process for a given sensor $v_{i}$ can be briefly described by Algorithm 1. Each sensor keeps a local data set $L_{i}$ to store the up-to-date data it receives and senses. For any data $d_{j}$ sensed or received by $v_{i}$, $d_{j}$ is first put into the local data set $L_{i}$ of $v_{i}$. Next, $v_{i}$ tries to fuse this data with relevant data in $L_{i}$ based on pre-selected fusion rules such as Bayesian inference method [27] and Dempster-Shafer theory [28]. The fusion rules are out of the scope of this paper, and so no explanation to that effect is provided. If the quality of these fused data is beyond the predefined threshold, $v_{i}$ will stop the propagation of this data and fuse them into a piece of valuable information $I_{h}$ with a credible confidence about a target $T_{k}$. Otherwise, $v_{i}$ will add the data into the pending queue $pendingQue_{i}$, and make communication decisions for all data in this queue.
**Algorithm 1** Distributed data relay process. 1: **while** true **do** 2:  $L_{i}\leftarrow$ data received or sensed by $v_{i}$ 3:  **for all** data $d_{j}$ received or sensed by $v_{i}$
**do** 4:   Try to fuse it with relevant data in $L_{i}$; 5:   **if** the quality of the fused data meets the threshold **then** 6:     Fuse them into a piece of information; 7:     Inform other nodes data $d_{j}$ is outdated; 8:   **else** 9:     $PendingQue_{i}\leftarrow d_{j}$;10:  Make communication decisions for each data in $PendingQue_{i}$;

The objective of data relay is to aggregate relevant data to some single node to fuse more high quality information while minimizing the energy consumption of the network. However, sensors cannot take optimal actions since they do not have global view of the network. They make data communication decisions based on local knowledge to maximize incremental quality of relevant data set of neighbors in local data set, and minimize the energy cost. For data $d_{j}$, there are two communication choices, broadcast $act_{v_{i}}^{d_{j}}=1$, or not $act_{v_{i}}^{d_{j}}=0$. If broadcasting, it is not absolutely sure that all the neighbors will receive this data because of the uncertainty of network connection. We can explain the objective function of data relay as following:

(1) $$\begin{array}{cc} \underset{act_{v_{i}}^{d_{j}}}{\mathrm{argmax}}act_{v_{i}}^{d_{j}} & \times (\sum_{v_{k}\in N\left(v_{i}\right)}P\left(v_{i},v_{k}\right)\times \Delta Q\left(d_{j},L_{k}\right)-\beta \times Energy\left(v_{i},d_{j}\right)) \end{array}$$

where $\Delta Q(d_{j},L_{k})$ is the incremental quality of knowledge base $L_{k}$ after receiving data $d_{j}$. $Energy(v_{i},d_{j})$ is the energy consumption on transmitting $d_{j}$, and *β* is a coefficient to balance the energy cost and information cost in decisions.

### 3.1. Information Quality

We use $L_{i}^{h}\subseteq L_{i}$ to express the subset of data that is relevant, and indicates information by $I_{h}$. The quality $Q(L_{i}^{h})$ of fused data in subset $L_{i}^{h}$ can be calculated based on the fusion rule of sensors. Let us take Dempster-Shafer rule [28] as an example,

(2) $$\begin{array}{c} Q\left(L_{i}^{h}\right)=\max_{T_{k}\in T}c_{L_{i}^{h}}\left(T_{k}\right)+c_{L_{i}^{h}}\left(CLUTTER\right) \end{array}$$

where $c_{L_{i}^{h}}\left(T_{k}\right)$ and $c_{L_{i}^{h}}\left(CLUTTER\right)$ are elements of $c_{L_{i}^{h}}=\left\{c_{L_{i}^{h}}\left(T_{1}\right),..,c_{L_{i}^{h}}\left(T_{M}\right),c_{L_{i}^{h}}\left(CLUTTER\right)\right\}$, representing the basic probability assignment for the fused data indicating information $I_{h}$. This can be calculated as $c_{L_{i}^{h}}={⊕}_{d_{k}\in L_{i}^{h}}c_{d_{k}}$, where “⊕” is the operator in D-S rule of combination. If the number of these data reaches the minimum number threshold $\omega_{n}$ and the quality of fused data is more than the minimum value threshold $\omega_{Q}$: $Q\left(L_{i}^{h}\right)>\omega_{Q}\;\&\left|L_{i}^{h}\right|>\omega_{n}$, these data will be fused into a piece of information $I_{h}$. The quality of the whole local data set is the sum of the quality of data sets:

(3) $$\begin{array}{c} Q\left(L_{i}\right)=\sum_{L_{i}^{h}\subseteq L_{i}}Q\left(L_{i}^{h}\right) \end{array}$$

As data is only fused with relevant data, adding one piece of data to a local dataset can only affect the quality of its related data set. The incremental quality of a database after receiving $d_{j}$ can be calculated as

(4) $$\begin{array}{cc} \Delta Q(d_{j},L_{i}) & =Q\left(L_{i}\cup \left\{d_{j}\right\}\right)-Q\left(L_{i}\right)=Q\left(L_{i}^{h}\cup \left\{d_{j}\right\}\right)-Q\left(L_{i}^{h}\right) \end{array}$$

Maintaining a precise value of the incremental quality based on Equation (4) for each neighbors is computational high. However, it is unnecessary to keep it so precise and an estimation of this value is enough for the relay decision. What we need is to obtain those data that have higher confidence and have more relevant data in the local data set. To simplify, we use the utility of a data set to approximately estimate the quality of same. The utility can easily be calculated as the sum of utility between data:

(5) $$\begin{array}{c} U\left(L_{i}\right)=1/2\sum_{d_{j}\in L_{i}}\sum_{d_{l}\in L_{i}}U\left(d_{j},d_{l}\right)\propto Q\left(L_{i}\right) \end{array}$$

where $U(d_{j},d_{l})$ is the utility between data. The value can be computed either according to the fusion rule of sensors, or given by an expert knowledge system. For example, utility can be looked up in a utility table stored in sensors as their domain knowledge. Particularly, $U(d_{j},d_{j})=0$, and if $d_{j}$ and $d_{l}$ are not relative, $U(d_{j},d_{l})=0$. Therefore the incremental quality can be approximately represented by the incremental utility:

(6) $$\begin{array}{ccc} \Delta Q(d_{j},L_{i}) & \propto \\ \Delta U(d_{j},L_{i}) & =U\left(L_{i}\cup \left\{d_{j}\right\}\right)-U\left(L_{i}\right) & =\left\{\begin{array}{cc} \sum_{d_{l}\in L_{i}}U\left(d_{j},d_{l}\right) & \mathrm{if}\;d_{j}\notin L_{k};\\ 0 & \mathrm{if}\;d_{j}\in L_{i} \end{array}\right. \end{array}$$

If $v_{i}$ already has observation about $d_{h}$, this data becomes redundant and makes no contribution to fusion, and as such, the benefit of broadcasting is 0. If $v_{i}$ does not have this data, it may be helpful to sensor $v_{i}$ to increase the fusion probability. The benefit in this case can be represented by the sum of utility between this data and other data in local data set.

### 3.2. Energy Consumption on Communication

In a deployed sensor network, the sensor nodes are usually battery powered [29], and they have to operate on an extremely frugal energy budget. Since communication is the major source of energy consumption in sensor networks, to prolong the lifetime of the sensor network requires a careful consideration of the energy cost in each transmission.

For each sensor $v_{i}$, the energy cost on communication is mainly composed of two parts: the energy of broadcasting data $d_{j}$: $Eb(v_{i},d_{j})$, and the energy of receiving data $d_{j}$: $Er(v_{i},d_{j})$. They can be computed as follows [24]:

(7) $$\begin{array}{cc} Eb(v_{i},d_{j}) & =\left(E_{elec}\left(v_{i}\right)+E_{amp}\left(v_{i}\right)\right)\times d_{j}.length\\ Er(v_{i},d_{j}) & =E_{elec}\left(v_{i}\right)\times d_{j}.length \end{array}$$

where $E_{elec}\left(v_{i}\right)$ is the energy consumed by $v_{i}$’s transmit electronics or receive electronics for digital coding, modulation and filtering of the signal, $E_{amp}\left(v_{i}\right)$ is the energy consumed by its TX amplifier, and $d_{j}.length$ (bits) is the length of data pieces. Suppose that the sensors are homogenous, and the size of data is identical. The energy cost of broadcasting and receiving a piece of data can be substituted by two constants Eb and Er respectively. The energy consumption on transmitting a piece of data is the sum of energy on broadcasting and all neighbors’ receiving:

(8) $$\begin{array}{c} Energy\left(v_{i},d_{j}\right)=Eb+Er\times \sum_{v_{j}\in N\left(v_{i}\right)}Pr\left(v_{i},v_{j}\right) \end{array}$$

## 4. Matrix-Based Data Relay Algorithm

In large-scale distributed sensor networks, because of the huge system size and energy constraints of sensors, sensors are unlikely to have a global observation to support optimal communication decisions. Sensors only make rational communication decisions soley based on their local dataset from their previously transmitted messages around the network. To make rational communication decisions, sensors need local topology of the network to indicate who are potential receivers, the local data distribution to indicate what data neighbors have, and the utility between data pieces to figure out the benefit of broadcasting one piece of data.

**Figure 2:**
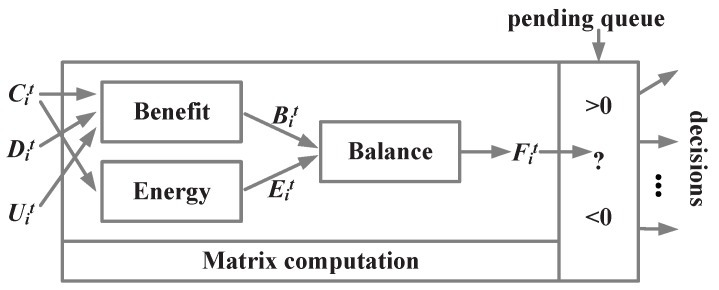
The frame of matrix-based Data Relay algorithm.

In this section, a Proactive Energy-Saving Data Relay algorithm CDU will be proposed to help sensors compute the benefit and cost for all the data in pendingQue by neat matrix computations. The framework is shown in Figure 2. First, sensors need local states to support their decisions. In our model, three parts are necessary to a sensor: connection matrix C denotes its local network topology, data distribution matrix D about the local data distribution of the sensor and its neighbors and the data Utility matrix U. For each time step *t*, each sensor *i* is required to update CDU matrixes by the model maintenance function introduced in the next section. Next, a neat computation with the CDU matrixes can produce the expected benefit of transmitting pending data $B_{i}^{t}$. In addition, connection matrix $C_{i}^{t}$ also be used to predict the cost of transmitting data $E_{i}^{t}$. By balancing between $B_{i}^{t}$ and $E_{i}^{t}$, if the sensor find that the expected utility $F_{i}^{t}$ is positive, it will broadcast the data as the network will be benefit of more likely fusing valuable information.

### 4.1. Basic Matrix Model

Before introducing these three matrices, we first need one data structure $N_{i}$ to store the sensors known by $v_{i}$, and it can be updated by the path of messages it receives. Since sensors have different local information, we take sensor $v_{i}$ as an example to describe these three matrices.

$C:1\times |N_{i}|\to \left[0,1\right]$ is the connection matrix to illustrate the connections between $v_{i}$ and other sensors recorded in $N_{i}$. Each element $C_{v_{i},v_{k}}$ represents the connection probability between $v_{i}$ and $v_{k}$ estimated by $v_{i}$. Especially, $\forall v_{i}\in V,\;C_{v_{i},v_{i}}=0$.

$D:|N_{i}\left|\times \right|L_{i}|\to \left[0,1\right]$ is the local data distribution matrix, and each element $D_{v_{k},d_{j}}$ shows the probability of $d_{j}$ in $v_{k}$’s local data set by $v_{i}$’s estimation.

$U:|L_{i}\left|\times \right|L_{i}|\to \left[0,c\right]$ is the utility matrix of data known by $v_{i}$, and each element $U_{d_{l},d_{k}}=U\left(d_{l},d_{k}\right)$ shows the incremental utility when adding $d_{l}$ meets $\left\{d_{k}\right\}$.

### 4.2. Benefit of Broadcasting

The benefit of broadcasting one piece of data $d_{j}$ is the sum of increased expected utility of all receivers. By multiplying Equation (1) by $\left(1-D_{v_{k},d_{j}}\right)$, the increased expected utility of $v_{k}$ receiving $d_{j}$ can be calculated in a unified manner by matrix computation.

(9) $$\begin{array}{cc} \Delta EU(d_{j},L_{k})= & \left(1-D_{v_{k},d_{j}}\right)\cdot \sum_{d_{l}\in L_{k}}U_{d_{h},d_{l}}\\ = & \left(1-D_{v_{k},d_{j}}\right)\cdot \sum_{d_{l}\in L_{i}}D_{v_{k},d_{l}}\cdot U_{d_{h},d_{l}}\\ = & \left(1-D_{v_{k},d_{h}}\right)\cdot \left(\vec{D_{v_{k},*}}\cdot \vec{U_{*,d_{h}}}\right) \end{array}$$

The benefit of broadcasting $d_{h}$ is the sum of increased information utility of sensors that can receive this data. However, for $v_{i}$, it does not know which sensor exactly receives this piece of data broadcast. Only the connection probability stored in matrix *C* indicates the probability of receiving this data. Therefore, the benefit of $v_{i}$ broadcasting $d_{h}$ can be computed as:

(10) $$\begin{array}{ccc} Benefit(v_{i},d_{h})= & \sum_{v_{k}\in N_{i}}C_{v_{i},v_{k}}\cdot \Delta EU\left(d_{h},L_{k}\right)= & C\cdot (\left(\vec{\Lambda }-\vec{D_{*,d_{k}}}\right)\circ \left(D\cdot \vec{U_{*,d_{k}}}\right)) \end{array}$$

where $D_{v_{k},*}$ is a row vector of matrix *D*, $\vec{\Lambda }={\left[1\right]}_{|N_{i}|\times 1}$ is a column vector of ones, the operator “∘” is the Hadamard product that takes two matrices with identical dimensions but only produces their corresponding elements. In matrix representation, one sensor can compute the benefit of broadcasting any of its data by a single matrix computation:

(11) $$\begin{array}{cc} B= & C\cdot ((\Gamma -D)\circ (D\cdot U)) \end{array}$$

where $B={\left[Benefit\left(v_{i},d_{h}\right)\right]}_{1\times |L_{i}|}$, and $\Gamma ={\left[1\right]}_{|N_{i}\left|\times \right|L_{i}|}$.

### 4.3. Energy Cost of Transmission

The energy cost of transmission is composed of two parts: cost of broadcasting, and cost of receiving. The broadcast energy cost of a piece of data is Eb. Before transmission, $v_{i}$ does not know exactly which sensor can receive this data, and can only estimate the whole receiving energy cost according to the connection probability in matrix $C_{i}$. The cost of $v_{i}$ transmitting $d_{h}$ can be estimated as follows,

(12) $$\begin{array}{ccc} Energy(v_{i},d_{h}) & =Eb+\sum_{v_{k}\in N_{i}}C_{v_{i},v_{k}}\cdot Er & =Eb+Er\cdot (C\cdot \vec{\Lambda }) \end{array}$$

Let matrix $E={\left[Energy\left(v_{i},d_{h}\right)\right]}_{1\cdot |L_{i}|}$, which represents the energy cost of $v_{i}$ for transmitting any data in $pendingQue_{i}$. Thus, this matrix can be calculated as follows:

(13) $$\begin{array}{c} E=\left(Eb+Er\cdot \left(C\cdot \vec{\Lambda }\right)\right)\cdot {\vec{\Lambda }}^{T} \end{array}$$

### 4.4. The Balance

To make communication decisions for each sensor data in pending queue, sensors balance the benefit of broadcasting with the energy cost as follows.

(14) $$\begin{array}{ccc} F= & B-\beta E= & C\times \left(\left(\Gamma -D\right)•\left(D\times U\right)\right)-\beta \cdot \left(Eb+Er\cdot \left(C\times \vec{\Lambda }\right)\right)\times {\vec{\Lambda }}^{T} \end{array}$$

where *F* is a $1\times |L_{i}|$ matrix and each element in *F* indicates the difference between the benefit and the energy cost of transmitting each data. Essentially, this can also be calculated in a single matrix computation.

In the decision process, for each data in $pendingQue_{i}$, if $F_{v_{i},d_{h}}>0$, which represents the benefit of broadcasting this data is bigger than the energy cost, this data is worth to be broadcast at this moment. Otherwise, it may not be broadcast. By ensuring a balance between increasing the quality of receivers and saving energy,

- the data that has higher confidence (that can make a higher contribution to fuse into an information) has a higher priority to be broadcast, which can avoid the energy consumption on unnecessary data retransmission.
- the data that is more relative to data of neighbors has a high probability to be broadcast. This guarantees that the related data are only transmitted in a small part of the whole network and aggregated toward some node rather than blind coverage.

## 5. Model Maintenance Algorithm

Sensors can make rational decisions by comparing the benefits and energy consumption of broadcast through matrix computation introduced in the previous section. Because of the mobility of sensors, and the dynamic changing nature of data distribution, the three matrices C,D and U need to be updated in time. The more precise the model is, the better the decisions. However, in large sensor networks, considering the massive energy cost on communication, no single sensor can get the precise and global connection and data distribution model but only partial observations.

In this section, considering the intrinsic energy cost in the operations of these networks, we propose heuristic updating approaches to maintain a local model for each sensor from its incoming messages. With these updating approaches, an integrated decision algorithm is considered to help each sensor make rational decisions with partial observations.

### 5.1. Initialization

Before deployment in large scale sensor networks, locations of sensors are not pre-determined, and the network topology is unknown to any sensor. Sat this stage, the three matrices of each sensor are initialized to empty matrices. After deployment, sensors start the introduction phase by broadcasting hello messages for once to initialize their connection matrix *C*.

### 5.2. The Rules to Maintain The Dimensions

As we described in Section 3, the size of these three matrices is related to $N_{i}$ and $L_{i}$. The column number of *C* and row number of *D* correspond to the size of $N_{i}$. The column number of *D* and the row and column number of *U* also correspond to the size of $L_{i}$. When an element is added into any of these two sets, one column or row of 0 will be added to the corresponding matrices. If one element is moved out of these sets, the corresponding column or row of matrices will be deleted. Therefore, the principles to maintain these two sets are paramount, and given below:

- $v_{i}$ will add an element $v_{j}$ in set $N_{i}$: when receiving a data $d_{k}$ and $v_{j}\in d_{k}.path$ is not in $N_{i}$.
- $v_{j}$ will be removed from $N_{i}$: when $v_{i}$ neither has positive connection probability with it nor has any knowledge of it, $C_{v_{i},v_{j}}$=0 and $\sum_{d_{h}\in L_{i}}S_{v_{j},d_{h}}$=0.
- $v_{i}$ will add an element $d_{k}$ into $L_{i}$:
  − when receiving data $d_{k}$ that is not in $L_{i}$.
  − when generating data $d_{k}$ based on its detection.
- $v_{i}$ will delete the element $d_{k}$ from $L_{i}$: when $d_{k}\in dataO$, where dataO stores data that has been fused or outdated.

### 5.3. Updating the Connection Matrix C

In wireless sensor networks, the connection matrix C is initialized by hello messages. However, because of the dynamically changing nature of the network topology, the connections between sensors may change. In this subsection, some heuristic rules are proposed to update matrix C from sensors’ incoming messages. When sensor $v_{i}$ receives a piece of data from sensor $v_{j}$, first, it indicates that $v_{j}$ is well connected with it:

$$C_{v_{i},v_{j}}=\sigma$$

where *σ* is a high probability that the two sensors are connected. Second, any two adjacent sensors in $d_{k}.path$ are well connected for their successful transmission in the last time step. The connection between them can be updated by $\sigma^{2}$ such that $\forall v_{j}\in d_{h}.path,\;v_{k}=d_{h}.path.next\left(v_{j}\right)$, and

$$C_{v_{j},v_{k}}=C_{v_{k},v_{j}}=\sigma^{2}$$

Also, $v_{i}$ will assume any connection probability in *C* fades as sensors move and failure of sensors occurs. This implies, the connection probability decays for a given period of time *T*. We can describe this process as follows:

C←C∘C

### 5.4. Updating the Data Distribution Matrix D

The better sensors have knowledge about the knowledge bases of their neighbors, the better decisions they can make. In this subsection, we focus on the data distribution updating approaches based on the data pieces sensors sense, broadcast and receive.

Algorithm 2 presents the updating process of data distribution matrix *D* for sensor $v_{i}$, where dataG, dataB, and dataR are data sets, which respectively store data sensed , data broadcast and data received by $v_{i}$ as well as outdated data. First, for each data generated by $v_{i}$ based on its own detection, obviously, it will update element $D_{v_{i},d_{h}}$ to 1 (line 1–2). Second, for each data broadcast by $v_{i}$, and for any neighbor $v_{j}$, the probability of receiving this data is $C_{v_{i},v_{j}}$. Therefore, according to the standard probability function, $v_{j}$’s probability of having data $d_{h}$ should be updated, $D_{v_{j},d_{h}}=1-\left(1-D_{v_{j},d_{h}}\right)\left(1-C_{v_{i},v_{j}}\right)$ (line 3–4). Third, for each data received by $v_{i}$, $v_{i}$, update $D_{v_{i},d_{h}}$ to 1 (line 6). Also, all nodes on $d_{j}.path$ have this data (line 8), and the nodes that are neighbors of nodes on the path have a probability of having this data, which can be calculated according to the standard probability function (line 9). Finally, for data, which relative information has been fused and outdated, its corresponding column in *D* should be deleted to save storage (line 10–11).
**Algorithm 2**
updateD(D,C,dataG,dataR,dataB). 1: **for all**
$d_{h}\in dataG$
**do** 2:   $D_{v_{i},d_{h}}\leftarrow 1;$ 3: **for all**
$d_{h}\in dataB$
**do** 4:   $\vec{D_{*,d_{h}}}\leftarrow \vec{\Lambda }-\left(\vec{\Lambda }-\vec{D_{*,d_{h}}}\right)\circ \left(\vec{\Lambda }-\vec{C_{v_{i},*}}\right)$;  5: **for all**
$d_{h}\in dataR$
**do** 6:   $D_{v_{i},d_{h}}\leftarrow 1$; 7:   **for all**
$v_{j}\in d_{h}.path$
**do** 8:    $D_{v_{i},d_{h}}\leftarrow 1$; 9:    $\vec{D_{*,d_{h}}}\leftarrow \vec{\Lambda }-\left(\vec{\Lambda }-\vec{D_{*,d_{h}}}\right)\circ \left(\vec{\Lambda }-C_{v_{j},*}\right)$;

### 5.5. Updating The Utility Matrix U

When generating or receiving a new piece of data, the utility between this data and the data in $L_{i}$ can be looked up in a table, which is stored as background knowledge before sensor $v_{i}$ was deployed according to their identity, location and timestamp. The corresponding elements in matrix *U* will then be updated. The details are shown as Algorithm 3. When receiving new data $d_{h}$, first, $v_{i}$ will judge if it is relative to the data in $L_{i}$(line 2,3) based on their timestamp and location. If that is the case, the utility between it and any relative data can be looked up in a table according to their identity (line 4). Else, the corresponding utility will be set to 0 since they indicate different information (line 6).
**Algorithm 3**
updateU(U,dataG,dataR,utilityTable). 1: **for all**
$d_{h}\in dataG\cup dataR$
**do** 2:  **for all**
$d_{j}\in L_{i};$
**do** 3:   **if**
$relative(d_{h},d_{j})$
**then** 4:    $U_{d_{h},d_{i}}\leftarrow utility\left(d_{h},d_{i},utilityTable\right);$ 5:   **else** 6:    $U_{d_{h},d_{i}}\leftarrow 0;$

The example below shows the update process of the utility matrix. At time *t*, the data set of $v_{1}$ is $L_{i}=\left\{d_{1}.d_{2}\right\}$, and the sensor set in the knowledge of $v_{1}$ is $N_{i}=\left\{v_{2},v_{3}\right\}$. The details of $d_{1},\;d_{2}$ are shown as follows:

$d_{1}=<v_{4},\left(T_{A},0.5\right)\left(T_{B},0.2\right)\left(CLUTTER,0.3\right),\left(1.1,2.1\right),2013/05/08/16:00,path=\left\{v_{4},v_{2}\right\}>,$

$d_{2}=<v_{7},\left(T_{A},0.4\right)\left(T_{B},0.5\right)\left(CLUTTER,0.1\right),\left(3.2,7.1\right),2013/05/08/13:00,path=\left\{v_{7},v_{3}\right\}>.$

As the location and timestamp of these two data pieces are different, they are not relevant, and matrix $U=\left(\begin{array}{ccc} & d_{1} & d_{2}\\ d_{1} & 0 & 0\\ d_{2} & 0 & 0 \end{array}\right)$. At time t+1, $v_{1}$ receives $d_{3}$ from $v_{3}$, and $d_{3}$ is shown in details as follows:

$d_{3}=<v_{8},\left(T_{A},0.6\right)\left(T_{B},0.3\right)\left(CLUTTER,0.1\right),\left(1.3,2.0\right),2013/05/08/16\;:\;03,path=\;\left\{v_{8},v_{5},v_{3}\right\}>.$

According to its location:(1.3,2.0) and timestamp:2013/05/08/16:03, which is close to $d_{1}$ but not $d_{2}$, $v_{1}$ can confirm that $d_{3}$ is related to $d_{1}$ but not $d_{2}$. It becomes obvious now that $U_{d_{3},d_{2}}=\;U_{d_{2},d_{3}}=\;0$. Right from here, $v_{1}$ will look up the utility between $d_{3}$ and $d_{1}$ in its reward table according to their identity.

- For target A, $c_{d_{1}}\left(T_{A}\right)=0.5$, $c_{d_{3}}\left(T_{A}\right)=0.6$. After checking utilitytable, $v_{2}$ can get $value(T_{A})=\;5.5$. Doing the same for target B, $v_{2}$ can get $value(T_{B})=3.5$
- The utility is a function of these two values. One possible way:
  $U_{d_{3},d_{1}}=value\left(T_{A}\right)+\omega \cdot value\left(T_{B}\right)=5.5+0.3\cdot 3.5=6.55$

Finally, the matrix $U=\left(\begin{array}{cccc} & d_{1} & d_{2} & d_{3}\\ d_{1} & 0 & 0 & 6.55\\ d_{2} & 0 & 0 & 0\\ d_{3} & 6.55 & 0 & 0 \end{array}\right)$ is obtained.

### 5.6. Integrated Algorithm

With these updating algorithms, network connection, data distribution and utility matrix can be updated to help sensors get local observations to support rational communication decisions even in dynamically changing networks. The whole data relay process with local knowledge can be seen in Algorithm 4.

This algorithm consists of two parts. In the first part, sensors update these three matrices based on the approaches mentioned in last three subsections (line 1–7). In the second part, $v_{i}$ will try to fuse with these new data and make communication decisions for those that have not been fused (line 8–21). Principally, $v_{i}$ checks them one by one to see if any new information can be fused (line 8–11). If the quality of the new information exceeds the predefined threshold, data related to this information will be fused and added into the outdated data set. $v_{i}$ will then inform other sensors to stop propagation of these outdated data pieces (line 11–13). If no information can be fused, this data will be added into the pending queue(line 15). After the information fusion process completes, $v_{i}$ makes decisions for data in pending queue based on our lightweight matrix computation (line 16) by balancing between the benefits and energy cost of broadcasting. If the benefit is higher than the energy cost (line 18), $v_{i}$ will add itself onto the path of this data and broadcast it (line 19–20), and update the data set dataB with this data. Otherwise, this data will be ignored (line 21).
**Algorithm 4** Data relay process for a sensor $v_{i}$. 1: **while**
true
**do** 2:  dataG←sensedData(); 3:  dataR←receivedData(); 4:  dataO←outdatedData() 5:  u pdateC(C,dataR); 6:  u pdateD(D,dataG,dataR,dataB,dataO); 7:  u pdateU(U,dataG,dataR,utilityTable); 8:  **for all**
$d_{h}\in dataG\cup dataR$
**do** 9:   **if**
$d_{h}\notin L_{i}$
**then**10:    $L_{i}\leftarrow L_{i}\cup \left\{d_{h}\right\}$;11:    **if**
$Quality\left(I_{j}|L_{i}\right)\geq \tau_{j})$
**then**12:     fuse into information $I_{j}$;13:     $dataO\leftarrow datarelatedtoI_{j}$;14:    **else**15:     $pendingQue.add(d_{h})$;16:  $F\leftarrow C\cdot \left(\left(\Gamma -D\right)\circ \left(D\times U\right)\right)-\beta \cdot \left(Eb+Er\left(C\cdot \vec{\Lambda }\right)\right)$;17:  **for all**
$d_{h}\in pendingQue$
**do**18:   **if**
$F(d_{h})>0$
**then**19:    $d_{h}.path.add\left(v_{i}\right)$;20:    $broadcast(d_{h})$;21:    $dataB.add(d_{h})$

## 6. Experimental Section

In this section, we evaluate the performance of our proactive data relay algorithm CDU through simulations. In most scenarios, we used a field size of 600 × 600 m${}^{2}$ where 500 mobile nodes were randomly scattered for target detection, and fused data detected into information. For each time step, 1% of sensors were made to move. Sensors communicated with each other in a broadcast medium, and the power of sensor radio transmitter was fixed so that any node within a 25 meter radius was within communication range. Sensor nodes within a communication range of another sensor are described as the neighbors. The power consumption (0.66 W in transmit mode, 0.395 W in receive mode) were chosen based on data from currently available radios [12]. The transmission time for a packet was fixed at 10ms. In each run, 100 events about targets randomly occurred. Each target could be detected by 9 sensors, and each detection generated a piece of data with a confidence *c*, c∈[0,1] is related to the distance between detected sensor and target [25]. For each event, one piece of information can be fused only when more than 6 data related to this target is aggregated by one sensor and the combined confidence is higher than 0.75.

We mainly compare the performance of our algorithm CDU with the Flooding [8], the Scalable Broadcast Algorithm (SBA) [14], and GRAdient Broadcast(GRAB) [12] algorithms. GRAB is on behalf of routing algorithms where only predefined sink nodes are able to fuse while the other three treat all sensors as potential sink nodes. Flooding is the most straightforward data relay algorithm, where each sensor broadcasts whatever new data it receives immediately without any reasoning. For SBA, only data that can reach new neighbors is broadcast. In SBA, neighbor knowledge in two hops is maintained by periodic "hello" packages. Also, in GRAB, each sensor maintains a cost field, and records the cost to the sink node in proportion to the distance to the sink node. Sensors only broadcast data received from the sensor where cost is higher and relay data to sink nodes to fuse. Typical of most simulations, the network has one random sink node. When the number of nodes is 1000, 2 sink nodes are randomly chosen. In CDU, Flooding, and SBA, any sensor can fuse data into information if enough data is aggregated, while in GRAB only sink node can fuse. The value of *U* matrix in CDU is generated based on the D-S fusion rule [30]. Other relevant parameters defined are σ=0.97,β=2.0,lengthOfPath=2.

To test if CDU works well, we measured the **Number of fused information** by all the sensors, the **Total energy cost** on communication of all sensors, and the **efficiency** = Number of fused information/Total energy cost, calculated as the energy consumed on communicating to fuse each piece of information. The efficiency indicates how the algorithms balance broadcasting to fusing more information and minimizing energy consumption. Results for each experiment are based on one hundred trials.

In the simulations, we first evaluate the impact of control parameters of CDU, including *β* and the length of path. Then we compare the performance of CDU with the other three related algorithms mentioned above in default settings with the progress of simulations. After that we compare these algorithms while independently changing the following environmental factors: the size of the field to test if CDU is scalable; the ratio of moved sensors to test if CDU can adapt to dynamic networks; and the threshold of fused targets. Finally, since GRAB is one of the routing protocols, we compare the final energy distribution between CDU and that of GRAB.

### 6.1. Different *β* and Different Length of Path

The value of *β* is a parameter used by CDU to balance forwarding data and minimizing energy consumption, which is defined in Equation (1). The path is a data structure used in CDU to store the sensor visited in each data. The length of path affects rich degree of the local matrix model.

**Figure 3:**
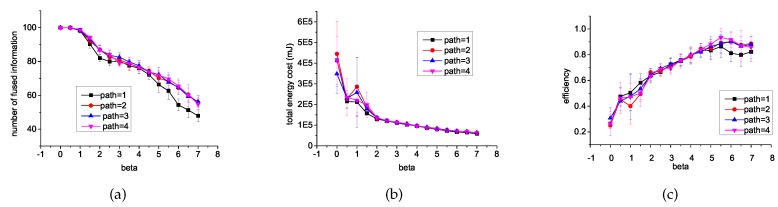
Experimental results with different *β* and different length of path. (**a**) introduces the comparisons of the number of fused information; (**b**) introduces the comparisons of the energy cost on communication; (**c**) introduces the comparisons of the fusion efficiency.

To understand how *β* and the length of path affect efficient data relay, we varied *β* from 0 to 4, and varied the length also from 1 to 4. Experimental results of a number of fused information, energy cost on communication, and efficiency are illustrated in Figure 3. As we can see, the higher the value of *β*, the definition of useless data becomes more strict, and more data will be stopped from broadcasting especially those data with lower confidence or well known by neighbors. To some degree, it affects the number of fused information. As shown in Figure 3a,b, the number of fused information is proportional to *β* and the energy cost decreases as *β* increases. When β=0, any data received will be broadcast and all the information can be fused. Figure 3c shows the efficiency for different *β*. When β=2.0, the efficiency reaches a high position. The balance of data relay and saving energy is good. When β>2.0, the efficiency still keeps a high value. However, the information fused is much less.

In the next simulations, the default value of *β* is 2.0. We notice that the performance of CDU is slightly affected by the length of the path. Regardless of the value of *β*, when the length of path is longer than 1, it performs better than when the length is 1. While the other values work almost the same for sensors, broadcast decisions can only affect their neighbors, and neighbors’ neighbors have higher probabilities to be neighbors than nodes far away with sensors moving around. However, the longer the path, the more storage space needed to store these matrices. Therefore in the following simulation, the default length of a path is 2.

### 6.2. Different Algorithms

**Figure 4:**
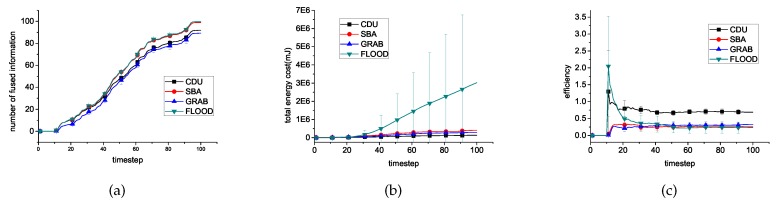
Experimental results for 500 sensors using different algorithms to fuse revelent data. (**a**) introduces the comparisons of the number of fused information. (**b**) introduces the comparisons of the energy cost on communication. (**c**) introduces the comparisons of the fusion efficiency.

In this experiment, we compared the performance of our data relay algorithm CDU, with the Flooding algorithm, SBA and GRAB. 100 events about targets randomly occurred during 10th–95th time steps. The results are shown in Figure 4. At the first 10 time steps, no events exist, and no information is fused as Figure 4a shows. During this period, sensors in SBA broadcast hello messages to maintain 2-hop neighbor knowledge [16] and sensors in GRAB maintain their cost field. Therefore, in Figure 4b the cost of these two algorithms is more than 0. In the next 90 time steps, as time goes by, information is fused as more and more energy is consumed on data relay.

In general, Flooding and SBA can fuse almost all the information. Correspondingly, their energy cost is higher than CDU because of flooding’s no limitations on broadcast and SBA’s purpose to cover all sensors with all data until it is possible to fuse them. GRAB fuse less information than CDU while consuming more for the long path to relay data to the fixed sink node. The efficiency of CDU is the highest of these algorithms for its effective constraining broadcast of the useless data.

### 6.3. Impact of Environmental Settings

#### 6.3.1. Network Size

**Figure 5:**
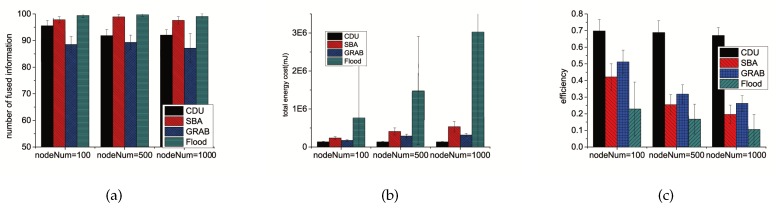
Experimental results with different network size using different algorithms. (**a**) introduces the comparisons of the number of fused information; (**b**) introduces the comparisons of the energy cost on communication; (**c**) introduces the comparisons of the fusion efficiency.

In this experiment, we studied how CDU scales to large networks for data relay. The number of sensors in the system are 100, 500 and 1000 while the side length of the squares are 250, 600, 850 to guarantee the same node density. The number of events is kept at 100. Figure 5 shows that regardless of the network size, CDU can well balance the broadcasting and saving energy, and its efficiency is always about 2 times as efficiency of the other algorithms.

#### 6.3.2. The Ratio of Moved Sensors

In this experiment, we evaluated the performance of these four algorithm to investigate if CDU can adapt to the dynamic network. We varied the move ratio of sensors at each time step from 0 to 0.22.

**Figure 6:**
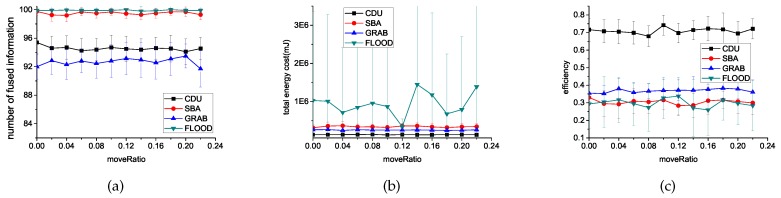
Experimental results with different move ratio using different algorithms. (**a**) introduces the comparisons of the number of fused information; (**b**) introduces the comparisons of the energy cost on communication; (**c**) introduces the comparisons of the fusion efficiency.

In SBA, sensors update two hop neighbor knowledge by periodic hello messages. For GRAB, sink nodes periodically broadcast advertisement messages, which help sensors update their cost field. However, in CDU , there is no periodic hello messages. A sensor will not broadcast hello message until it moves. Figure 6 shows that the performance of these four algorithms is unaffected by the variation of move ratio and CDU is always way ahead in efficiency.

#### 6.3.3. Threshold

We first varied the minifusedNum from 3 to 8, while using a fixed 0.75 minifusedConfidence. Then, we varied the minifusedConfidence from 0.4 to 0.85 while using a fixed 6 minifusedNum threshold.

**Figure 7:**
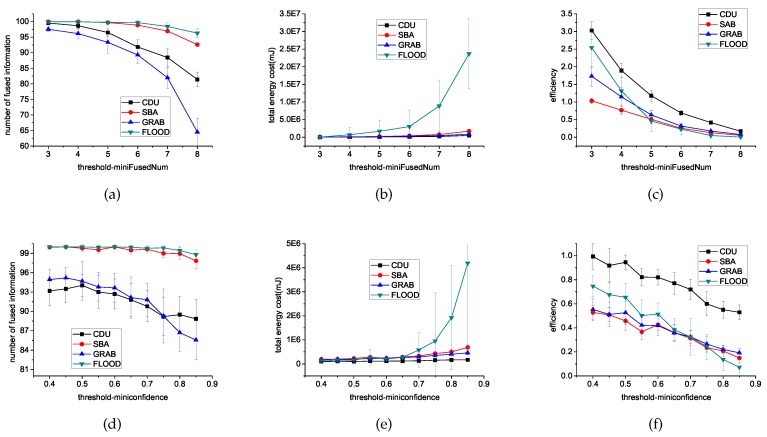
Experimental results for different threshold using different algorithms. (**a**) introduces the comparisons of the number of fused information with minifusedNum from 3 to 8; (**b**) introduces the comparisons of the energy cost on communication with minifusedNum from 3 to 8; (**c**) introduces the comparisons of the fusion efficiency with minifusedNum from 3 to 8; (**d**) introduces the comparisons of the number of fused information with minifusedNum from 0.4 to 0.85; (**e**) introduces the comparisons of the energy cost on communication with minifusedNum from 0.4 to 0.85; (**f**) introduces the comparisons of the fusion efficiency with minifusedNum from 0.4 to 0.85.

Figure 7 shows that when the threshold of minifusedNum or minifusedConfidence is higher, less information can be fused in the same 100 time steps. However, more energy consumption and lower efficiency on data relay for a sensor is needed to aggregate more data to reach the number and confidence threshold. Figure 7c,f show CDU has the highest efficiency of all these algorithms.

### 6.4. Energy Distribution

**Figure 8:**
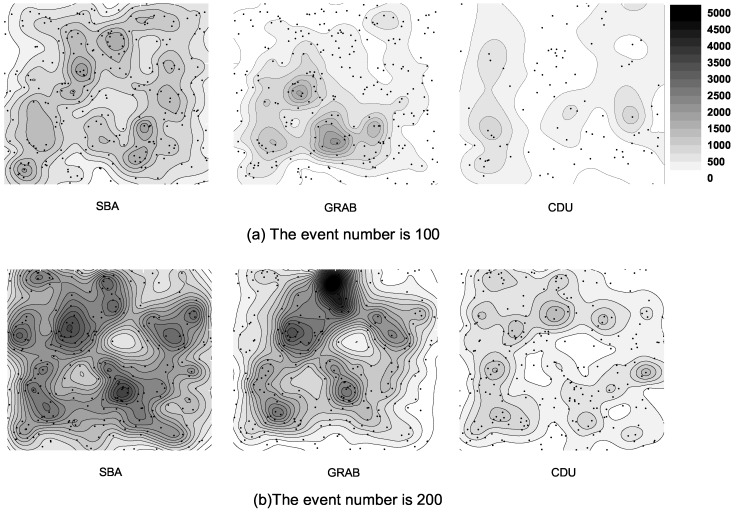
Energy consumption distribution(mJ) for different scenarios. (**a**) introduces the comparisons of the energy distribution after relaying data with 100 events; (**b**) introduces the comparisons of the energy distribution after relaying data with 200 events.

In this subsection, we compare the energy distribution between CDU and SBA, and the routing protocol GRAB. Figure 8 shows the final energy distribution after relaying data for 100 and 200 events while the network size is 500. As shown in these figures, the three algorithms all consume more energy in Figure 8b, where more events happened than in Figure 8a. In SBA and CDU, energy consumption is better distributed than in GRAB. For GRAB, data should be relayed to sink node to fuse and sink nodes, and nodes near sink nodes distinctly consume more energy. However, in SBA, sensors try to cover every node with data until fused, and the energy is much more than CDU.

## 7. Conclusions

Large scale multi-sensor fusion is a very important issue in future networks and internet of things, especially in the domains of disaster response and military operations. However, previous centralized data aggregation algorithms for small sensor network are no longer feasible in considering of huge network expenses as well as the energy expenses for central nodes. In this paper, we have proposed an extremely economic data relay algorithm that sensors could proactively make broadcast decisions by neat matrix computation to balance transmission and save energy. By encoding sensors’ local knowledge to three matrixes: network connection, data distribution and data utility matrix, they can do the reasoning for all the pending data by only a neat matrix computation. We also built heuristic algorithms for sensors to well maintain those matrixes with only a local view to the network so that this design can be adaptive to the scalable and dynamic environment. Our experimental simulations manifested that our approach is scalable and effectively balance between promoting data fusion process and saving energy to prolong the life time of the whole network.

## Figures and Tables

**Table 1:** An example of the body of a piece of data.

Target	Confidence	Target Type	Confidence
USSR T80	0.4	US M977	0.001
WSSR T72M	0.3	US M35	0.001
US M1	0.1	US AVENGER	0.001
US M1A1	0.05	US HMMWV	0.001
USSR 2S6	0.02	USSR SA9	0.001
USSR ZSU23	0.03	CLUTTER	0.095
